# Educational innovation: mobile applications as complementary tools in the training of plastic surgery residents

**DOI:** 10.1590/acb411926

**Published:** 2026-04-17

**Authors:** André Luiz Monteiro dos Santos Marins, Matheus Galvão Valadares Bertolini Mussalem, Antônio de Pádua Peppe, José da Conceição Carvalho, Lydia Masako Ferreira

**Affiliations:** 1 Universidade Federal de São Paulo – Escola Paulista de Medicina – Disciplina de Cirurgia Plástica – São Paulo (SP) – Brazil.

**Keywords:** Education, Medical, Graduate, Internship and Residency, Mobile Applications, Smartphone, Educational Technology, Surgery, Plastic

## Abstract

**Purpose:**

To identify and describe mobile applications that can serve as complementary tools in the education of plastic surgery residents.

**Methods:**

A structured literature review was conducted across MEDLINE, EMBASE, ERIC, Latin American and Caribbean Health Sciences Literature (LILACS), and Cumulative Index to Nursing and Allied Health Literature (CINAHL) databases. Search strategies used Medical Subject Headings (MeSH) terms and relevant keywords. Additional searches were performed in the App Store and Google Play to identify mobile applications related to plastic surgery education. Inclusion criteria covered studies and applications directly linked to residency training in plastic surgery.

**Results:**

Fourteen articles met eligibility criteria, and 18 mobile applications specifically related to plastic surgery education were identified. The majority was freely available and addressed topics such as surgical simulation, flap design, anatomy, and procedural guidance. Apps in English predominated across platforms.

**Conclusion:**

Mobile applications represent valuable complementary resources for plastic surgery training, supporting both theoretical learning and technical development. Their structured integration into residency curricula could enhance academic and clinical performance among residents.

## Introduction

The widespread use of smartphones and tablets has transformed the learning environment in plastic surgery residency programs, facilitating rapid access to theoretical and procedural knowledge^1-3^. This technological integration assists residents in overcoming challenges inherent to surgical education and the adoption of new medical techniques^3-5^. Mobile applications particularly promote continuous and self-directed learning, enabling residents to review anatomy, watch procedural videos, and access scientific databases from any location^6-10^.

During the SARS-CoV-2 pandemic, these digital tools became crucial for maintaining training continuity despite the suspension of elective procedures^3,11^. Nevertheless, they are designed to complement—not replace—practical surgical experience, serving to reinforce motivation, confidence, and skill acquisition^2,12^. Despite the growing number of plastic surgery–related apps, many are aimed at marketing or patient engagement rather than structured resident education^3,4,13^.

Although more than 325,000 healthcare applications exist, few undergo usability testing or validation, which limits their reliability for formal training^14,15^. Considering the reduced operative exposure and working-hour restrictions in surgical residencies, virtual learning environments have become vital adjuncts for developing technical proficiency^4,16,17^. The Brazilian plastic surgery curriculum emphasizes comprehensive clinical competence, yet national literature assessing the integration of digital tools remains limited^18,19^.

In other surgical specialties, educational mobile platforms such as the Electronic Neurosurgical Register and Zwisch Me have successfully enhanced feedback and operative assessment^20,21^. Inspired by these advances, this study aimed to identify and describe mobile applications that can complement plastic surgery residency education, outlining their main features and potential contributions to surgical learning.

## Methods

### Study design

A structured literature systematic review and complementary mobile application search were conducted to identify digital tools that support plastic surgery residency education. The study followed descriptive and observational methodology, focusing on the characterization of available resources and their educational potential.

### Eligibility criteria

Articles published in Portuguese, English, or Spanish addressing plastic surgery residency education, surgical simulation, learning curves, or mobile applications were included. Exclusion criteria comprised studies unrelated to surgical education, reports on other medical specialties, editorials, letters, case reports, and COVID-19-specific clinical training papers. Duplicates were removed before screening.

### Search strategy

The literature search was performed on October 3, 2024, using the databases MEDLINE, EMBASE, ERIC, Latin American and Caribbean Health Sciences Literature (LILACS), and Cumulative Index to Nursing and Allied Health Literature (CINAHL). Search strategies incorporated Medical Subject Headings (MeSH) and free-text terms related to “plastic surgery,” “residency,” and “mobile applications.” The full search strings are presented in Table 1. Two independent reviewers conducted the selection process, and discrepancies were resolved by consensus with a third reviewer.

**Table 1:** Search strategies.

Database	Search strategy	Results
EMBASE	(('handheld' OR 'handhelds') AND 'application':ti,ab,kw OR 'portable application':ti,ab,kw OR 'mobile application'/exp OR 'smartphone*' OR 'mobile platform':ti,ab,kw OR 'mobile solution':ti,ab,kw) AND ('general surgery'/exp OR 'plastic surgery'/exp) AND ('clinical competence'/exp OR 'decision support system'/exp OR 'cognition'/exp OR 'mnemonics':ti,ab,kw OR 'checklist'/exp OR 'metacognition':ti,ab,kw OR 'thinking'/exp OR 'thinking':ti,ab,kw OR 'memory'/exp OR 'cognition':ti,ab,kw OR 'skill acquisition':ti,ab,kw OR 'diagnostic reasoning':ti,ab,kw OR 'feedback system'/exp OR 'clinical decision making'/exp OR 'decision making'/exp OR 'transcultural care'/exp OR 'education'/exp OR 'professional competence'/exp OR 'social competence'/exp) AND [embase]/lim	159
MEDLINE	((("handheld"[All Fields] OR "handhelds"[All Fields]) AND "application"[Title/Abstract]) OR "portable application"[Title/Abstract] OR "mobile applications"[MeSH Terms] OR "smartphone*"[All Fields] OR "mobile platform"[Title/Abstract] OR "mobile solution"[Title/Abstract]) AND ("general surgery"[MeSH Terms] OR "plastic surgery procedures"[MeSH Terms]) AND ("clinical competence"[MeSH Terms] OR "decision support techniques"[MeSH Terms] OR "Cognition"[MeSH Terms] OR "mnemonics"[Title/Abstract] OR "checklist"[MeSH Terms] OR "metacognition"[Title/Abstract] OR "thinking"[MeSH Terms] OR "thinking"[Title/Abstract] OR "memory"[MeSH Terms] OR "Cognition"[Title/Abstract] OR "skill acquisition"[Title/Abstract] OR "diagnostic reasoning"[Title/Abstract] OR "feedback"[MeSH Terms] OR "clinical decision making"[MeSH Terms] OR "decision making"[MeSH Terms] OR "culturally competent care"[MeSH Terms] OR "educational measurement"[MeSH Terms] OR "professional competence"[MeSH Terms] OR "social skills"[MeSH Terms])	46
CINHAL	( (skill OR clinical competence or clinical skills or clinical competency) AND ("mobile app" OR "mobile application" OR "handheld application" OR smarthphon*) ) AND residents	27
LILACS	(medico* OR resident*) AND (habilidad* OR "competencia clinica" OR aprendizag*) AND ("tecnologia da informação" OR "aplicativos moveis" OR "mobile App" OR smarthphon*) AND ( db:("LILACS"))	47
ERIC	("decision making" OR learning OR aptitude OR "cognitive processes" OR "knowledge level" OR mnemonics OR recall OR "individual development" OR skills OR competence OR "difficulty level" OR expertise) AND resident* AND ("mobile app" OR smarthphon* O "mobile platform" OR "handheld application")	50

CINHAL: Cumulative Index to Nursing and Allied Health Literature; LILACS: Latin American and Caribbean Health Sciences Literature.

Source: Elaborated by the authors.

### Endpoints

The primary endpoint was the identification and qualitative description of mobile applications applicable to plastic surgery residency training. Secondary endpoints included classification of journals by impact factor, distribution of applications by cost, and language availability.

### Mobile application search

In June 2024, complementary searches were conducted on the App Store and Google Play platforms using keywords such as “plastic surgery training app,” “microsurgery education,” and “flap surgery simulation.” Applications were included if they offered educational, procedural, or anatomical content related to plastic surgery training.

### Data extraction and analysis

Relevant data from included studies and applications were extracted into structured tables summarizing titles, objectives, outcomes, and educational relevance. Impact factors were categorized as low (0.50–1.067), medium (1.068–13.975), or high (13.976–51.568) according to Paiva et al.^22^. Descriptive analysis was performed to interpret the distribution of findings across categories.

## Results

**Figure 1:**
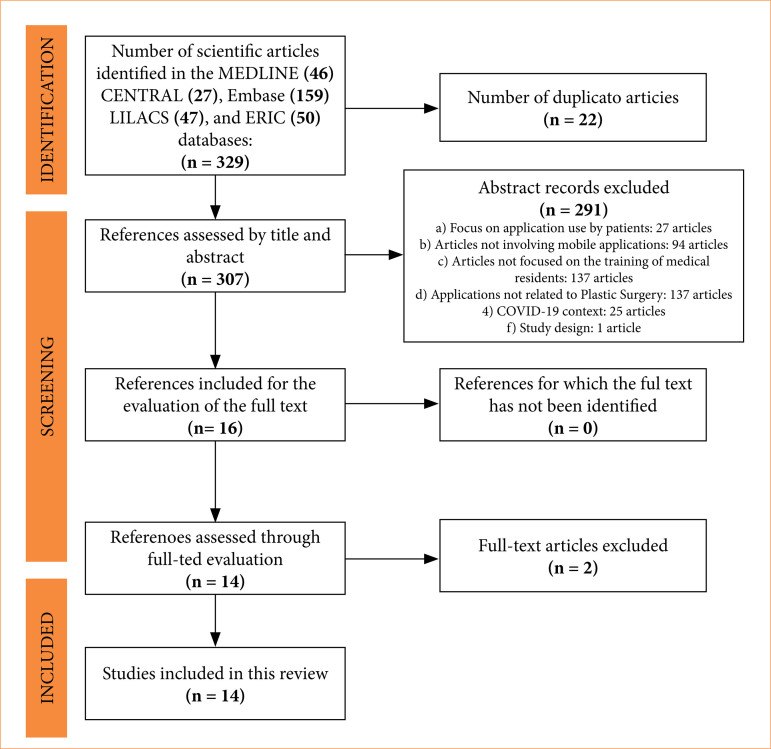
PRISMA flow diagram.

CINHAL: Cumulative Index to Nursing and Allied Health Literature; LILACS: Latin American and Caribbean Health Sciences Literature. Source: Elaborated by the authors.

A total of 329 articles were identified across the five databases. After removing 22 duplicates, 307 articles were screened by title and abstract. Following the application of the inclusion and exclusion criteria, 14 studies were included in the final analysis (Fig. 1). The PRISMA flow diagram (Fig. 1) summarizes the selection process.

### Characterization of included studies

**Figure 2:**
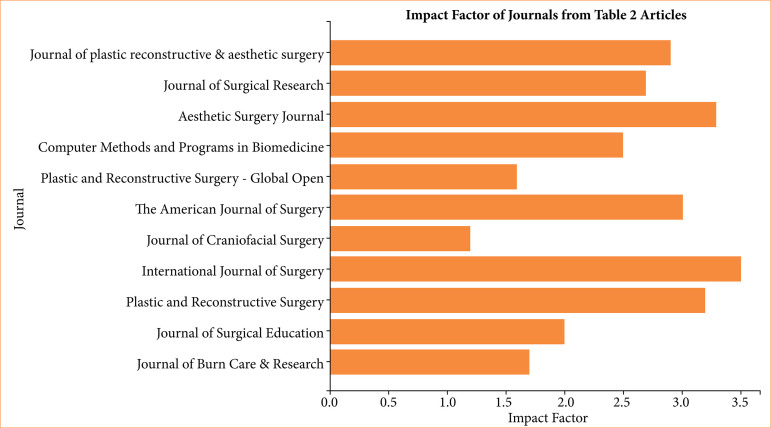
Classification of articles according to journal and impact factor.

The selected studies were published between 2011 and 2024, primarily originating from the United States of America and the United Kingdom (Table 2). Journals varied in impact factor, with a mean of 2.714—classified as moderate according to Paiva et al.^22^. Most studies focused on the use of digital tools or mobile applications in surgical education, particularly for improving operative performance, anatomical understanding, and procedural confidence (Table 3). Figure 2 illustrates the distribution of publications according to journal impact factor.

**Table 2:** Classification of articles according to title, author, year, country of origin, and publishing journal.

Title of the article	Authors	Year	Country	Journal
“The rise of technology in plastic surgery education: is the textbook dead on arrival (DOA)?”^1^	Waltzman et al.	2016	United States of America	*Aesthetic Surgery Journal*
“A scoping review of mobile apps in plastic surgery: patient care, trainee education, and professional development”^3^	JARVIS et al.	2023	United States of America	*Plastic and Reconstructive Surgery Global Open*
“Creation of an application to increase surgical resident operative case coverage”^5^	Robbins et al.	2023	United States of America	*Journal of Surgical Education*
“Maximizing technological resources in plastic surgery resident education”^7^	Khansa and Janis	2015	United States of America	Journal of Craniofacial Surgery
“Uniting evidence-based evaluation with the ACGME plastic surgery milestones”^12^	Kobraei et al.	2016	United States of America	Plastic and Reconstructive Surgery
“Smartphones and the plastic surgeon”^16^	Al-Hadithy and Ghosh	2013	United Kingdom	Journal of Plastic, Reconstructive & Aesthetic Surgery
“Smartphone applications for the plastic surgery trainee”^23^	Amin	2011	United Kingdom	Journal of Plastic, Reconstructive & Aesthetic Surgery
“iGuide to plastic surgery: iPhone apps, the plastic surgeon, and the health care environment”^24^	Mohan and Branford	2012	United States of America	Aesthetic Surgery Journal
“An interactive augmented reality software for facial reconstructive surgeries”^25^	Guo et al.	2024	China	Computer Methods and Programs in Biomedicine
“The future of burns surgery training—using handheld technology”^26^	Walsh et al.	2018	United Kingdom	Journal of Burn Care & Research
“Implementation of an online intraoperative assessment of technical performance for surgical trainees”^27^	Faber et al.	2023	United States of America	Journal of Surgical Research
“The reliability of resident self-evaluation of operative performance”^28^	Kendrick et al.	2021	United States of America	American Journal of Surgery
“Smartphone applications in plastic surgery: a cross-sectional survey”^29^	Grow et al.	2019	United States of America	Aesthetic Surgery Journal
“Live Surgery: An innovative plastic surgery teaching programme for medical students utilising real-time operating theatre audiovisual link-ups”^30^	Smeeton et al.	2019	United Kingdom	Journal of Plastic, Reconstructive & Aesthetic Surgery

Source: Elaborated by the authors.

**Table 3:** Classification of articles according to title, study aim, results, and conclusions.

Title	Study objective	Results	Conclusions
“The rise of technology in plastic surgery education: is the textbook dead on arrival (DOA)?”^1^	To explore the rise of technology in plastic surgery education and the impact on traditional textbooks.	86.5% of residents own iPhones and 90% own tablets (mainly iPads). 42% of residents use the Plastic Surgery Education Network (PSEN), an online learning platform that offers surgical videos and educational modules, weekly. However, 78% of the residents have never used the RADAR Resource, an application focused on aesthetics, which offers videos, case studies, and discussion forums. Sixty percent still use physical books weekly.	Technology will be crucial in the future of plastic surgery education. The integration of platforms like iPhone and iPad can provide rapid access to large volumes of information. Program directors should support the use of technology, but physical books still play an important complementary role.
“A scoping review of mobile apps in plastic surgery: patient care, trainee education, and professional development”^3^	To review the use of mobile applications in plastic surgery, including education, patient care, and professional development.	Among the 80 publications analyzed, 20 were included. The applications were categorized into three main areas: patient care and surgical applications, professional development and education, and marketing and practice development. The mean evidence score was 4.2 according to the American Society of Plastic Surgeons Evidence Rating.	Mobile applications are valuable resources for patients, surgeons, and trainees, facilitating care, communication, and professional development. However, the full impact of these applications on patient decision-making and expectations requires further investigation.
“Creation of an application to increase surgical resident operative case coverage”^5^	To create an application to increase the operative case coverage of surgical residents.	The volume of operative cases covered increased significantly after the implementation of the application, with improvements in the coverage of endoscopic, laparoscopic, open, and robotic cases (p < 0.001). One hundred percent of the residents reported better knowledge of available cases and simplification in the search for coverage.	The application showed a positive impact on the operative experience of residents and can be used to improve educational opportunities in surgical training programs nationwide.
“Maximizing technological resources in plastic surgery resident education”^7^	To maximize the use of technological resources in the education of plastic surgery residents.	The PSEN was identified as one of the most useful resources, offering surgery videos, simulations, interactive modules, and courses with specialists. PSEN allows the creation of personalized learning plans and is used by more than 90 residency programs, facilitating resident learning and preparation.	The integration of technological resources into the residency curriculum is essential to optimize resident learning, improving surgical preparation and efficiency in surgical practices. The use of videos, simulators, and video feedback can accelerate the development of surgical skills.
“Uniting evidence-based evaluation with the ACGME plastic surgery milestones”^12^	To unite evidence-based evaluation with the ACGME milestones in the evaluation of plastic surgery residents.	The System for Improving and Measuring Procedural Learning (SIMPL) application was used in 20 cases, allowing for quick and detailed assessments of performance and autonomy. Data collection took less than 1 minute per case, and the average time for submitting the assessment was 5 hours. SIMPL provided specific and concrete data for each procedure.	SIMPL facilitates the continuous and detailed collection of data on resident performance in each procedure. This tool has the potential to improve Milestone evaluations and provide quality feedback on resident progression over time. If widely adopted, it can reduce the administrative burden and improve competency-based training.
“Smartphones and the plastic surgeon”^16^	To explore the use of smartphones in the practice of plastic surgeons.	Smartphones are accessible and portable tools for education, telemedicine, and clinical practice. Useful applications include tridimensional anatomical guides, burn calculators, and surgery simulators. In telemedicine, the use of smartphones to monitor free flaps and burn diagnosis was effective, with 76% agreement compared to in-person assessments.	Smartphones democratize access to education and clinical care, especially in resource-limited areas. They have the potential to improve efficiency in patient care, but concerns about confidentiality and the quality of some applications need to be addressed.
“Smartphone applications for the plastic surgery trainee”^23^	To discuss the use of smartphone applications for the training of plastic surgery residents.	Scarcity of applications aimed at reconstructive plastic surgery training, with most available focused on the cosmetic market.	There is a need for the development of specific educational applications for plastic surgery, addressing anatomy, surgical techniques, and trauma management, with attention to the ethical issues of data confidentiality.
“iGuide to plastic surgery: iPhone apps, the plastic surgeon, and the health care environment”^24^	To discuss the use of iPhone applications for plastic surgeons in the healthcare environment.	Applications divided into four main categories: marketing, education, clinical tools, and communication. It was identified that 81% of physicians in the United States of America would use smartphones by 2012. The review found more than 10,000 medical and health applications, with only a limited number (less than 5%) focused specifically on plastic surgery.	Smartphones have great potential to improve medical practice, but there is a need for the development of more applications aimed at plastic surgery, especially for educational and clinical purposes.
“An interactive augmented reality software for facial reconstructive surgeries”^25^	To develop an interactive augmented reality software for facial reconstructive surgeries.	The application accurately overlaid relaxed skin tension lines in over 91% of cases across 263 facial images. A Monte Carlo experiment with simulated users was conducted to objectively assess performance, and the results followed a normal distribution.	The application teaches the fundamentals of facial reconstructive procedures, enabling interactive learning and objective performance assessment of trainee surgeons, particularly in aligning skin flaps with tension lines.
“The future of burns surgery training—using handheld technology”^26^	To discuss the future of burns surgery training using handheld technology.	Thirty-eight trainees participated. The group with no prior burns experience (group 1) increased the average score from 30 to 72%. The group with four to six months of experience (group 2) improved from 45.8 to 72.4%, and the group with 12 to 18 months of experience (group 3) increased from 85 to 100%.	The application demonstrated a significant educational benefit for burn surgery simulation, being the first tool of its kind to be applied on smartphones. Handheld technology offers an innovative method for students and surgeons to learn and improve surgical practices in burns.
“Implementation of an online intraoperative assessment of technical performance for surgical trainees”^27^	To implement and evaluate an online system for intraoperative assessment of surgical resident technical performance.	Around 560 evaluations were completed for 56 trainees supervised by 122 faculty members. The average number of procedures evaluated per resident was 10 and per evaluator was 4.6. The tool reliably differentiated residents by level of seniority based on technical and non-technical performance.	The online technical assessment system initiated by residents is viable and scalable, ensuring that feedback is delivered directly to trainees. The economical implementation via cloud computing services facilitates the adoption of this model by other training programs.
“The reliability of resident self-evaluation of operative performance”^28^	To evaluate the reliability of resident self-evaluation in surgeries using the SIMPL tool to compare with the evaluations of their preceptors.	Among 7,382 evaluations, 46٪ showed discrepancy between the residents' self-evaluations and the preceptors' evaluations, with 80٪ of residents underestimating themselves. The level of disagreement increased as residents advanced in training.	Resident self-evaluation is often inaccurate, with many underestimating their skills. This poses a challenge to the professional regulation of surgeons, and interventions to improve residents' ability to accurately self-evaluate are needed before independent practice.
“Smartphone applications in plastic surgery: a cross-sectional survey”^29^	To conduct a cross-sectional survey on the use of smartphone applications in plastic surgery.	Around 155 relevant applications were identified. Almost 100% of the 577 respondents own smartphones, and 90.49% of participants use smartphones weekly for medical purposes. Only 8.41% of the respondents knew more than 20 relevant applications.	Smartphone use is almost universal among plastic surgeons, but awareness of available applications is limited. There is a gap between the availability of applications and professionals' knowledge of these tools, suggesting that smartphones are not being used to their full potential in education and clinical practice.
“Live Surgery: An innovative plastic surgery teaching programme for medical students utilising real-time operating theatre audiovisual link-ups”^30^	To evaluate the use of LiveSurgery as an educational tool for medical students, connecting anatomy classes and surgical procedures in real-time.	Participants rated the experience with an average of 9/10 for educational utility and 8.5/10 for interest. Ninety percent of students found it easy to ask questions via interactive links during the live surgery.	LiveSurgery is a valuable tool for demonstrating plastic surgical procedures interactively. It has the potential to teach students and residents a variety of simple and complex plastic surgeries, being a promising innovation in medical education.

Source: Elaborated by the authors.

### Identification of educational mobile applications

**Figure 3:**
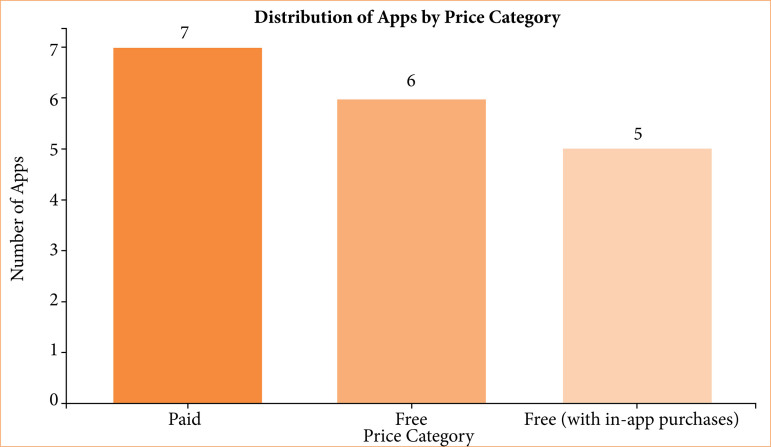
Classification of mobile applications according to pricing category.

Source: Elaborated by the authors.

Eighteen mobile applications relevant to plastic surgery education were identified through searches on the App Store and Google Play (Table 4). Most of these applications were designed to facilitate procedural learning, simulation, and anatomical visualization. Thematically, they covered areas such as flap design, microsurgical training, burn management, craniofacial surgery, and aesthetic planning. Figure 3 presents the distribution of applications according to the pricing model: six (33.3%) were paid, seven (38.9%) were free, and five (27.8%) were free with in-app purchases.

**Table 4:** Supportive apps for plastic surgery residency available for download.

Name	Price	Language	Description
iFlap	Paid	English	Clinical solutions for reconstructive surgery problems, presenting 86 flap surgeries through animations, videos, and tips.
Surgical Flaps	Paid	English	Tridimensional illustrations and textual explanations of various surgical flap techniques.
Flaps (Plastic Surgery Key)	Free	English	Microsurgical flap techniques, including procedural steps, anatomical considerations, and management strategies.
TeachMeSurgery	Free (with in-app purchases)	English	Covers a range of surgical topics, including flaps and skin grafts, with detailed classifications, indications, and step-by-step guides.
Surgical Flaps by Matt Briggs	Paid	English	Describes techniques for reconstructing cutaneous defects in plastic surgery, using tridimensional illustrations and animations.
Zwisch Surgery	Paid	English	Provides surgical skills assessment for residents and surgeons.
SIMPL OR	Free	English	Real-time surgical skills assessment and feedback during operations.
Academia de Especialistas	Paid	Portuguese	Offers courses with video lessons, training, and a question bank for preparation for the specialist title exam in Plastic Surgery in Brazil.
iNova Plástica	Free (with in-app purchases)	Portuguese	Teaching platform with video lessons and training on plastic surgery.
Gray's Anatomy — Atlas	Paid	English, French, German, Italian, Japanese, Chinese, and Spanish	Includes complete tridimensional models of the human body for the study of anatomy, as well as detailed views of key organs at various levels.
Breast Advocate	Free	English	Shared decision-making application for breast surgery and reconstruction. Offers personalized evidence-based recommendations, considering the patient's diagnosis and preferences. It is developed to assist in choosing the best treatment options.
Breast Augmentation Planner	Free (with in-app purchases)	English	Assists in planning breast augmentations, allowing help in choosing implants and pre-operative markings. Includes illustrative videos and a step-by-step interface to facilitate the planning process.
Stanford Microsurgery and Resident Training	Free	English	Application for microsurgery training for residents and trainees, offering a review of the components of microsurgery. Includes information for developing skills outside the surgical environment, helping users gain confidence and competence to work in the operating room.
Mobile eLogbook	Free	English	Assists surgeons in recording and tracking their surgical activities, including details of procedures, hospitals, and consultants, with automatic synchronization with the online platform elogbook.org

Source: Elaborated by the authors.

### Language distribution and accessibility

**Figure 4:**
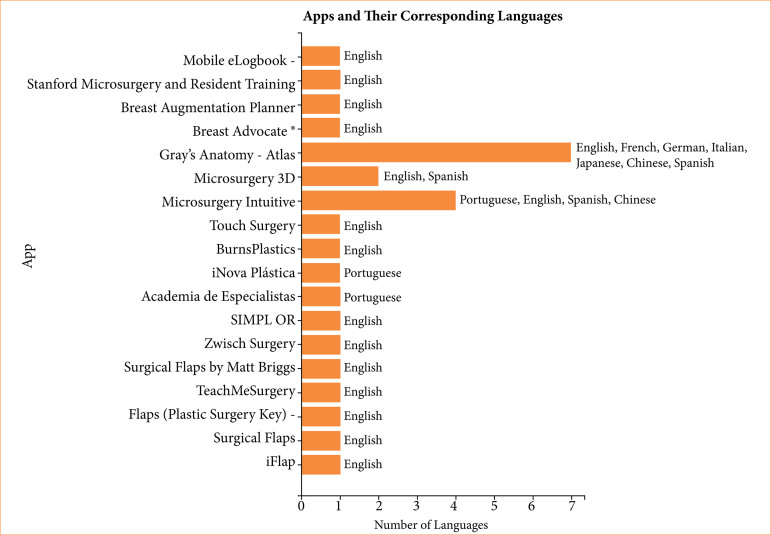
Mobile applications and their available language options.

Source: Elaborated by the authors.

Figure 4 shows that most applications were available in English, underscoring the predominance of English-language resources in surgical education. Only a small proportion of apps offers multilingual support, highlighting an opportunity for localization and accessibility improvements.

#### Summary of findings

Overall, the analysis revealed that freely available applications are the most common, indicating efforts to expand educational access without imposing financial barriers. However, the paid applications often featured advanced modules and higher interactivity. The consistent presence of moderate-impact journals among the included studies reflects the emerging—but still consolidating—nature of mobile technology research in surgical education. Together, these findings suggest that mobile applications are progressively being integrated into plastic surgery residency training as effective adjuncts for knowledge reinforcement and procedural practice.

## Discussion

Technological innovation has reshaped surgical education by introducing mobile applications that extend learning opportunities beyond traditional settings^23,24^. In this review, 18 applications were identified, most of which were free and designed to improve access to procedural guidance, anatomical visualization, and theoretical content. The predominance of freely available tools suggests an ongoing effort to democratize educational access while maintaining cost-effectiveness for residents.

The average journal impact factor of 2.714 observed among the included studies indicates that research on mobile education in surgical training is gaining academic recognition but remains in development. This reflects a growing global interest in technological integration within residency programs^31^. Journals such as *Plastic and Reconstructive Surgery* and *Aesthetic Surgery Journal* have played key roles in disseminating evidence-based innovations related to simulation and mobile learning.

Previous studies have shown that mobile tools enhance residents’ preparedness and confidence before operative experiences. Khansa and Janis^7^ emphasized the relevance of surgical simulators, online platforms, and video-based assessments as complementary tools that shorten the learning curve and foster skill refinement. Similarly, Waltzman et al.^1^ demonstrated that while most residents possess smartphones, few fully utilize specialized applications, underscoring the need for institutional guidance and structured integration into curricula. Grow et al.^29^ corroborated these findings, revealing high device ownership but limited awareness of educational applications, particularly among residents and fellows.

These data highlight an important gap between availability and utilization. The effective incorporation of mobile technologies into residency curricula requires deliberate planning, emphasizing usability, validation, and alignment with learning objectives. Institutions should encourage the use of peer-reviewed applications that provide reliable content and measurable educational outcomes^25-27^. As augmented and virtual reality technologies evolve, future tools may offer even greater interactivity and realism, further enhancing surgical training^17,26^.

Although the findings of this review indicate promising educational potential, they also underscore the scarcity of validated, specialty-specific resources in plastic surgery. Continuous evaluation and adaptation of mobile platforms are essential to ensure that these tools truly complement hands-on learning. Future research should focus on assessing objective outcomes such as knowledge retention, technical performance, and user’s satisfaction to consolidate the role of digital education in surgical training.

## Conclusion

Mobile applications represent effective complementary tools in plastic surgery residency training. The identified apps provide accessible resources for theoretical learning, anatomical visualization, and procedural guidance, directly supporting residents’ educational needs. Despite their availability, structured curricular integration and validation remain limited. Encouraging the adoption of reliable, evidence-based tools can enhance learning efficiency and technical development, contributing to more comprehensive and modern surgical education.

## Data Availability

The data will be available upon request.
